# Platelet Measurements and Type 2 Diabetes: Investigations in Two Population-Based Cohorts

**DOI:** 10.3389/fcvm.2020.00118

**Published:** 2020-07-10

**Authors:** Benjamin A. T. Rodriguez, Andrew D. Johnson

**Affiliations:** The Framingham Heart Study, Population Sciences Branch, Division of Intramural Research, National Heart Lung and Blood Institute, Framingham, MA, United States

**Keywords:** diabetes mellitus, platelets, platelet aggregation, mean platelet volume (MPV), sex differences, metabolic syndrome

## Abstract

Type 2 diabetes is a major risk factor for cardiovascular disease. Given the contribution of platelets to atherothrombosis—which in turn is a major contributor to cardiac events, there may be cause to consider platelet function in management of diabetes. Despite the large body of research concerning the role of platelets in cardiovascular complications of type 2 diabetes, evidence from population-based studies of platelet aggregation in diabetes is limited. Mean Platelet Volume (MPV), a cell trait partially associated with markers of platelet activity, is more commonly available. We investigated the association of metabolic syndrome and diabetes with platelet aggregation to three physiological agonists, ADP, collagen, and epinephrine, in the Framingham Heart Study Offspring cohort. We further examined the relationship between MPV measured with Beckman Coulter LH750 instruments and self-reported diabetes as well as MPV and diabetes medication in the UK BioBank cohort, performing the largest such analysis to date. Increased platelet aggregation associated with prevalent diabetes was observed for low concentration epinephrine (0.1 μM) alone and only in analyses of participants stratified either by male sex and/or having metabolic syndrome. Other agonists and concentrations were not significant for prevalent diabetes, or in opposite direction to the main hypothesis (i.e., they showed lower platelet aggregation associated with diabetes). After a median of 18.1 years follow-up, no platelet aggregation trait was associated with increased risk of diabetes (*n* = 344 cases). As expected, increased MPV was significantly associated with diabetes (β = 0.0976; *P* = 8.62 × 10^−33^). Interestingly, sex-stratified analyses indicated the association of MPV with diabetes is markedly stronger in males (β = 0.1232; *P* = 1.00 × 10^−31^) than females (β = 0.0514; *P* = 7.37 × 10^−5^). Among diabetes medications increased MPV was associated with Insulin (β = 0.1341; *P* = 1.38 × 10^−11^) and decreased MPV with both Metformin (β = 0.0763; *P* = 1.99 × 10^−6^) as well as the sulphonylureas (β = 0.0559; *P* = 0.0034). Each drug showed the same direction of effect in both sexes, however, the association with MPV was nearly twice as great or more in women compared to men. In conclusion, platelet function as measured by aggregation to ADP, collagen, or epinephrine does not appear to be consistently associated with diabetes, however, MPV is robustly associated suggesting future work may focus on how MPV segments pre-diabetics and diabetics for risk prediction.

## Introduction

Platelets play a central role in the development of atherothrombosis, a major contributor to cardiovascular events (1). Type 2 diabetes is a major risk factor for cardiovascular disease (2) and a strong predictor of cardiovascular mortality (3). Metabolic syndrome, a cluster of lipid, and nonlipid risk factors of metabolic origin (4), is prevalent and associated with an increased risk for diabetes and CVD in both sexes (5). Multiple aspects of atherothrombosis are suspected to be dysregulated in diabetes, including increased atherosclerosis, chronically activated endothelium, coagulation, and platelet reactivity (6). Platelet activation has been associated with progressive thickening of the carotid artery in diabetic patients (7). Given the contribution of platelets to atherothrombosis there may be cause to consider platelet function in management of diabetes and its macrovascular complications, but this is not part of the current standard of care (8).

Platelets in patients with diabetes are reported to exhibit hyper-reactivity to subthreshold stimuli, and undergo rapid consumption, resulting in accelerated thrombopoiesis of more reactive platelets (9). Reports of increased platelet aggregation to various agonists go back several decades (10, 11); however, studies in diabetics without CVD have produced inconsistent results (12–16). Platelet function in metabolic syndrome is less well-characterized. The two previous studies of platelet aggregation comparing metabolic syndrome patients with healthy controls produced inconsistent results (17, 18). Alterations in several platelet functional measures have been reported in both diabetes and metabolic syndrome: increased platelet P-selectin (17, 19), increased levels of thromboxane metabolites (18, 20), and increased platelet turnover (17, 21). Mean Platelet Volume (MPV), a measurement of platelet size, is partially associated with other established markers of platelet activity, including platelet aggregation (1, 22). Elevated MPV is associated with diabetes (23–27) and has been shown to be an independent prognostic marker of vascular events in diabetics (28) as well as the general population (29).

The majority of previous studies concerning platelet aggregability in diabetes or metabolic syndrome have been of limited sample size and often lacked adjustment for potential confounders known to affect platelet aggregation independent of these conditions, including age and sex. Platelets are significantly more reactive at baseline and after aspirin therapy in women compared to men (30). The relationship of sex with platelet aggregation in diabetes is largely unknown. To this end, we investigated the association of platelet aggregation to three physiological agonists with metabolic syndrome and diabetes in the FHS Offspring cohort, accounting for sex in our models and further stratifying analyses by sex where possible. We further examined the relationship between MPV with self-reported diabetes, accounting for sex, in the UK BioBank cohort, performing the largest such analysis to date. Finally, we examined the relationship between MPV and diabetes medication status, also accounting for sex.

## Methods

### Framingham Heart Study (FHS) Cohort, Phenotypes, and Analysis

The FHS is a longitudinal community-based cohort (31). This investigation considered participants in the Framingham Offspring cohort attending the fifth examination cycle (1991–1995) during which platelet function was assayed. Of 3,799 individuals attending the exam, 551 were excluded for one of the following reasons: missing platelet function measures mainly due to the measures being started partway in the exam cycle (*n* = 526), missing current diabetes status (*n* = 2), currently taking an antiplatelet medication other than aspirin (*n* = 14), previous history of leukemia (*n* = 3) or lymphoma (*n* = 7). The Institutional Review Board of Boston University Medical Center (Boston, MA) approved the study protocol, and all participants provided written informed consent.

Platelet aggregation was tested in response to three agonists: ADP (Sigma–Aldrich), epinephrine (Sigma–Aldrich), and collagen (Bio/Data), and arachidonic acid (Bio/Data). Blood samples were collected from the antecubital vein in the morning with participants lying supine after an overnight fast. Blood was drawn into an evacuated collection tube containing 3.8% sodium citrate (Becton Dickinson) and centrifuged at 160 g for 5 min at room temperature to separate platelet rich plasma. Blood was at RT until assay. Platelet rich plasma was not diluted to any specific concentration before assay. A 4-channel light transmission aggregometer (Bio/Data, Horsham, PA) was used to measure platelet aggregation at 37°C and 1,200 rpm. Aggregation was tested with a fixed concentration of arachidonic acid (1.64 mM) and increasing concentrations of ADP (0.05–15 μmol/L) and epinephrine (0.01–15 μmol/L) (32). As concentrations for epinephrine and ADP were titrated up or down based on individual responsiveness, not all participants were tested at all concentrations. Collagen lag time was measured in response to a single concentration (1.9 μg/mL) of calf-derived Type 1 fibrillar collagen. An increase in collagen lag time is indicative of reduced platelet aggregation. For ADP and epinephrine maximal aggregation at each concentration normalized relative to platelet poor plasma was used (scale of 0–100%). All reactions were run the same morning as the blood draw. Aspirin usage was inferred on the basis of the absence of full response to arachidonic acid stimulation. This was done by lab technician opinion after several minutes of aggregation (33).

Because of the variable number of participants tested at a given concentration of agonist, we focused our continuous trait analyses on two concentrations of ADP (1 and 5 μM) and epinephrine (0.1 and 1 μM) which had a sample size >900 at each concentration of agonist. We further characterized the association of prevalent diabetes with platelet aggregation by dichotomous aggregation traits previously applied in the analysis of venous thrombosis and CVD (32, 34). Briefly, ADP hyperreactivity was defined as ≥50% maximal aggregation to at least one low concentration (0.05, 0.1, 0.5, and 1 μmol/L). Similarly, hyperreactivity to epinephrine required ≥50% maximal aggregation to at least one low concentration (0.05, 0.1, 0.5, and 1 μmol/L). In contrast, those who failed to reach 50% maximal aggregation at a higher concentration (5, 10, and 15 μmol/L) of ADP or epinephrine were considered hyporesponders. Histograms of maximal percent aggregation at concentrations used to determine hyperreactivity and hyporeactivity are shown for ADP in Supplementary Figure 1 and for epinephrine in Supplementary Figure 2.

Criteria for diabetes mellitus were a fasting glucose level of ≥126 mg/dL, non-fasting glucose level ≥200 mg/dL, or use of medications to treat hyperglycemia. No specific steps were taken to exclude type 1 diabetes. Metabolic syndrome status was determined by the unified criteria of the International Diabetes Federation Task Force on Epidemiology and Prevention, National Heart, Lung, and Blood Institute, American Heart Association, World Heart Federation, International Atherosclerosis Society, and International Association for the Study of Obesity (35). The presence of any three of five risk factors constitutes a diagnosis of metabolic syndrome. The risk factors and their cut points are: elevated waist girth (male, ≥94 cm; female, ≥80 cm), elevated triglycerides (≥150 mg/dL), reduced HDL (male, <40 mg/dL; female, <50 mg/dL), elevated blood pressure (systolic ≥130 and/or diastolic ≥85 mm Hg), elevated fasting glucose (≥100 mg/dL) (35).

In this study, there were 1,143 distinct family clusters that contributed data. For a reference point, only 45 families out of 1,143 had family size >10 members, so the vast majority of families in the Gen2 study reflect sibships and close cousins. In the FHS cohort multivariable linear mixed-effect models correcting for nonindependence of families were used to determine the association of continuous platelet aggregation traits with metabolic syndrome and/or prevalent diabetes. Similarly, the association of incident diabetes with continuous traits was determined by Cox mixed-effect models. Duration of follow-up was calculated as days between date of baseline examination with no diabetes (Exam 5) and date of the examination where the participant first met the criteria for diabetes (up to Exam 9), ranging from 2.5 to 22.5 years. The association of prevalent diabetes with dichotomized platelet aggregation traits was assessed by multivariable logistic regression analyses. All models adjusted for age at baseline, sex, and aspirin usage at baseline (as determined by low PRP platelet aggregation to 5mg/mL arachidonic acid). A two-tailed *P* < 0.05 was considered nominally significant. Statistical analyses were performed with R, version 3.41. For additional sensitivity analyses, we stratified participants based on aspirin usage (yes/no), or history of cardiovascular disease (yes/no) as previously defined. Main models for prevalent diabetes were already adjusted for age at baseline, sex, and aspirin usage. We ran additional sensitivity models adding history of CVD as a further covariate.

### UK BioBank Cohort, Phenotypes, and Analysis

The UK Biobank is a large population-based prospective study (*n* = 500,000), established to allow investigations of the genetic and nongenetic determinants of the diseases of middle and old age (36). IRB approval was granted by the North West Haydock Research Ethics Committee (16/NW/0274) and this project approved by application to the UK Biobank. Participants were assessed between 2006 and 2010 in 22 locations throughout the UK. The assessment visit comprised electronic signed consent, a self-completed touch-screen questionnaire, a computer-assisted interview, physical, and functional measures, as well as collection of blood, urine, and saliva (36). Blood samples from participants were collected into 4 mL EDTA vacutainers by vacuum draw at assessment centers and stored at 4°C. Samples were transported overnight to the UK Biocenter (Stockport, UK) in temperature-controlled containers where full blood cell counts were measured using four Beckman Coulter LH700 Series hematology analyzers (37). The blood cell count values were QC'd centrally by the UK BioBank before release. We applied exclusion criteria that included (A) missing values for any of the following: MPV measurement, blood pressure, BMI, self-report of diabetes diagnosed by a physician, use of anti-platelet, blood pressure, or hyperlipidemia medication; (B) self-report of any of the following conditions: pregnancy, cancer drug treatments, malignant lymph nodes, bone metastases, leukemia, lymphoma, myelodysplasia, multiple myeloma; (C) ICD9 and/or ICD10 codes for any of the following conditions: HIV, neoplasms of bone, polycythaemia vera, myelodysplastic syndrome, red cell aplasia, anemias, coagulation defects, other diseases of the blood, diseases of the liver, abnormal findings on blood tests, splenectomy, and hematopoietic stem cell transplantation. A total of 36,297 participants were removed after applying the exclusion criteria. Among those remaining samples with MPV measures, we used the UK BioBank code (2443) indicating participant self-report of diabetes having been diagnosed by a physician. Use of anti-platelet (aspirin, clopidogrel, and dipyridamole), blood pressure, hyperlipidemia, or diabetes medications (insulin, metformin, and sulphonylureas) was also based on self-report. We defined hypertension as a systolic blood pressure ≥ 130 mm Hg, a diastolic blood pressure ≥ 85 mm Hg, or use of any antihypertensive medication. The association of MPV with self-reported diabetes or with diabetes medications among self-reported diabetics was assessed by multivariable linear analyses. No specific steps were taken to exclude type 1 diabetes. All models adjusted for age at baseline, sex, BMI, hypertension, antiplatelet medication, and hyperlipidemia medication. Secondary analyses were performed using interaction terms to assess effect modification by sex. A two-tailed *P* < 0.05 was considered nominally significant. Statistical analyses were performed with R, version 3.41.

## Results

### Framingham Heart Study

The study sample consisted of 3,244 FHS participants from the Offspring cohort who attended Exam 5 (here treated as baseline) and underwent platelet function testing. Platelet aggregation data from different agonists were available for epinephrine (*n* = 3,086), ADP (*n* = 3,215), and collagen (*n* = 3,146). This is the largest population-based study of platelet aggregation by LTA, the gold standard assay in the field, published to date, and to our knowledge the second largest (*n* = 244 here vs. *n* = 257 previously) such cross-sectional study conducted in diabetics (38). We believe this is the largest prospective study of platelet aggregation and diabetes (*n* = 344 cases). Descriptive statistics for the population demographics at the baseline examination are shown according to diabetes status in Table 1. The average participant age was 55.0 years (SD, 9.9 years) and the sample was 52.8% women (*n* = 1,715). At the baseline exam 41% (*n* = 131) met the criteria for metabolic syndrome (≥3 of 5 traits) and 7.5% (*n* = 244) were classified as having diabetes. In linear mixed effects models examining either sex or age separately along with family structure but no other covariates, female sex was strongly associated with higher platelet reactivity for all tests, and weaker associations were observed with older age being associated with increased platelet reactivity (except for collagen lag time; Supplementary Table 1). Hence all models, except those sex stratified, adjusted for both age and sex.

**Table 1:** Baseline characteristics of Framingham participants.

	**Diabetic** **(*****n*** **=** **244)**		**Not diabetic** **(*****n*** **=** **3,004)**		
**Baseline characteristics**	**Mean (n)**	**SD (%)**	**Mean (n)**	**SD (%)**	***P*-value**
Female (%)	94	38.5	1,621	54	4.68E-06
Age, year	60.3	8.7	54.6	9.9	2.20E-19
BMI, kg/m^2^	31.16	5.75	27.14	4.84	3.95E-22
Metabolic syndrome	217	88.9	1,114	37.1	4.96E-56
Fasting glucose, mg/dL	178.74	62.69	95.25	9.7	1.86E-55
HDL, mg/dL	40.72	12.19	50.57	15.18	5.60E-27
LDL, mg/dL	123.43	33.001	126.78	32.81	0.146
TRIG, mg/dL	225.53	156.16	143.05	101.46	1.98E-14
SBP, mmHg	139.39	19.72	125.39	18.71	1.20E-22
DBP, mmHg	74.21	10.02	78.02	10.25	5.37E-08
Waist girth, in	41.25	5.33	36.31	5.51	5.99E-34
Hypertension medication	87	41.8	481	16.9	1.31E-19
Lipid medication	34	16.3	177	6.2	2.88E-08
Aspirin usage	70	28.7	440	14.6	9.62E-09
Diabetes medication	117	48	0	0	n.a.
Insulin, *n* (%)	39	15.9	n.a	n.a.	n.a.
Oral hypoglycemic, *n* (%)^*^	88	36.1	n.a.	n.a.	n.a.

*BMI indicates body mass index; HDL, high-density lipoprotein; LDL, low-density lipoprotein; SBP, systolic blood pressure; DBP, diastolic blood pressure; n, sample size; SD, standard deviation; P-value, univariate T-test P-value*.

* *sub-drug not specified but in the time period of this Exam from January 1991 to June 1995 TZDs had not been released and metformin was only released in 1995 so all or nearly all of these participants were likely treated with sulfonylurea drugs*.

We first investigated the relationship between platelet reactivity and metabolic syndrome at the baseline examination. Results of the linear mixed-effect models are presented in Table 2. Reduced platelet aggregation in response to 5 μM ADP (β = 3.07; *P* = 1.7 × 10^−5^), 0.1 μM (β = 2.92; *P* = 0.032), and 1 μM epinephrine (β = 3.11; *P* = 0.011) as well as increased collagen lag time (β = 2.07; *P* = 0.015) were associated with metabolic syndrome after adjusting for age, sex, and aspirin therapy. Analyses stratified by sex showed aggregation was uniformly reduced across agonists and concentrations in females (four of five traits *P* < 0.05), but not in males (one of five traits *P* < 0.05). Further, the magnitudes of the associations with metabolic syndrome were greater in females compared to males (Table 2). The same patterns emerge when diabetics with metabolic syndrome are excluded from the analyses (Supplementary Table 2), suggesting the association is a pre-diabetic phenomenon. In the absence of metabolic syndrome, by contrast, female sex (compared to male sex) is associated with strong, uniform increases in aggregation across agonists and concentrations (five of five traits *P* < 0.05; Supplementary Table 3).

**Table 2:** Results of linear mixed-effect models for association of metabolic syndrome with platelet aggregation and stratified by sex.

					**Males**				**Females**			
**Phenotype**	**Beta**	**SE**	***P***	***N***	**Beta**	**SE**	***P***	***N***	**Beta**	**SE**	***P***	***N***
1 μM ADP	0.05	0.78	9.46E-01	2,999	1.04	0.93	2.63E-01	1,353	−1.14	1.22	3.52E-01	1,646
5 μM ADP	−3.07	0.71	1.70E-05	2,069	−2.66	1.05	1.16E-02	1,051	−3.77	0.95	6.78E-05	1,018
0.1 μM EPI	−2.92	1.37	3.26E-02	1,560	0.03	1.89	9.87E-01	609	−5.18	1.90	6.38E-03	951
1 μM EPI	−3.11	1.23	1.15E-02	2,485	−1.66	1.84	3.67E-01	1,134	−4.78	1.65	3.67E-03	1,351
COLLAGEN	2.07	0.85	1.54E-02	3,144	1.07	1.25	3.96E-01	1,473	3.47	1.16	2.82E-03	1,671

*Sample size (N) at different concentrations vary due to concentration titrating scheme originally applied in FHS LTA data acquisition. ADP and EPI values are % Maximal Aggregation whereas COLLAGEN is Lag time. Main model adjusts for age, sex, aspirin usage. Sex stratified model adjusts for age, aspirin usage. SE, standard error; P, P-value*.

We next investigated the relationship between platelet reactivity and prevalent diabetes (including those with metabolic syndrome). Results of the linear mixed-effect models are presented in Table 3. After adjusting for age, sex, and aspirin therapy we observed that reduced platelet aggregation in response to 5 μM ADP (β = 4.55; *P* = 7.5 × 10^−4^), 1 μM epinephrine (β = 7.89; *P* = 7.0 × 10^−4^) as well as increased collagen lag time (β = 8.04; *P* = 1.8 × 10^−7^) were associated with diabetes status. These are the same three traits and directions of effect as we observed in metabolic syndrome (Table 2). There was a trend toward increased aggregation to 0.1 μM epinephrine. Stratifying the analysis of 0.1 μM epinephrine by sex, it was evident the increased reactivity to epinephrine associated with diabetes was limited to males (β = 9.75; *P* = 0.006) and absent in females (β = 0.41; *P* = 0.928). The association in response to 1 μM ADP differed between the sexes with aggregation increasing in males and decreasing in females, but the models did not reach statistical significance. As previously observed in the models of metabolic syndrome, platelet aggregation measures in females again showed stronger negative associations with diabetes compared to males (Table 3). The interpretation of significance and directions of effects were not altered if, instead of adjusting for aspirin use, we stratified to non-aspirin or aspirin takers (Supplementary Table 4). Likewise, if analyses were adjusted for or stratified based on prior CVD history they remained largely unchanged (Supplementary Table 5).

**Table 3:** Results of linear mixed-effect models for association of prevalent diabetes with platelet aggregation and stratified by sex.

					**Males**				**Females**			
**Phenotype**	**Beta**	**SE**	***P***	***N***	**Beta**	**SE**	***P***	***N***	**Beta**	**SE**	***P***	***N***
1 μM ADP	0.01	1.45	9.93E-01	2,998	2.58	1.60	1.06E-01	1,352	−4.02	2.48	1.06E-01	1,646
5 μM ADP	−4.55	1.35	7.51E-04	2,068	−3.91	1.92	4.17E-02	1,050	−5.70	1.88	2.48E-03	1,018
0.1 μM EPI	5.27	2.93	7.23E-02	1,560	9.75	3.58	6.40E-03	609	0.41	4.55	9.28E-01	951
1 μM EPI	−7.89	2.33	7.04E-04	2,485	−5.44	3.36	1.05E-01	1,134	−10.98	3.23	6.72E-04	1,351
COLLAGEN	8.04	1.54	1.81E-07	3,144	6.35	2.08	2.29E-03	1,473	10.85	2.34	3.50E-06	1,671

*Sample size (N) at different concentrations vary due to concentration titrating scheme originally applied in FHS LTA data acquisition. ADP and EPI values are % Maximal Aggregation whereas COLLAGEN is Lag time. Main model adjusts for age, sex, aspirin usage. Sex stratified models adjusts for age, aspirin usage. SE, standard error; P, P-value*.

We explored the relationship between aggregation and diabetes further in multivariable analyses accounting for metabolic syndrome. Results of the linear mixed-effect models are presented in Table 4. There was a stronger association with increased aggregation to 0.1 μM epinephrine (β = 6.84; *P* = 0.022) compared to the diabetes model without metabolic syndrome as a covariate (Table 3). Reduced aggregation in response to 5 μM ADP (β = 3.42; *P* = 0.013) and 1 μM epinephrine (β = 6.88; *P* = 0.004), as well as increased collagen lag time (β = 7.58; *P* = 1.7 × 10^−6^) remained significantly associated with diabetes status. Stratifying the multivariable analyses by sex again showed increased reactivity to 0.1 μM epinephrine was associated with diabetes in males alone (β = 10.06; *P* = 0.006). Females, by contrast, once again showed stronger negative associations of platelet aggregation measures with diabetes compared to males (Table 4). In this multivariable model the repressive effect of metabolic syndrome on three measures: 0.1 μM (β = 5.26) and 1 μM epinephrine (β = 3.48) as well as 5 μM ADP (β = 3.48) remained significant in females independent of diabetes whereas in males only 0.1 μM epinephrine (β = 2.37) remained significant (data not shown).

**Table 4:** Results of multivariable linear mixed-effect models for association of prevalent diabetes with platelet aggregation including metabolic syndrome status and stratified by sex.

					**Males**				**Females**			
**Phenotype**	**Beta**	**SE**	***P***	***N***	**Beta**	**SE**	***P***	***N***	**Beta**	**SE**	***P***	***N***
1 μM ADP	−0.01	1.50	9.94E-01	2,998	2.31	1.64	1.58E-01	1,352	−3.66	2.58	1.56E-01	1,646
5 μM ADP	−3.42	1.38	1.34E-02	2,068	−3.10	1.95	1.13E-01	1,050	−3.89	1.95	4.65E-02	1,018
0.1 μM EPI	6.84	2.99	2.21E-02	1,560	10.06	3.65	5.77E-03	609	3.66	4.68	4.34E-01	951
1 μM EPI	−6.88	2.40	4.08E-03	2,485	−5.01	3.43	1.44E-01	1,134	−9.08	3.36	6.97E-03	1,351
COLLAGEN	7.58	1.59	1.75E-06	3,144	6.26	2.13	3.30E-03	1,473	9.65	2.43	7.24E-05	1,671

*Sample size (N) at different concentrations vary due to concentration titrating scheme originally applied in FHS LTA data acquisition. ADP and EPI values are % Maximal Aggregation whereas COLLAGEN is Lag time. Main model adjusts for age, sex, aspirin usage, and metabolic syndrome status. Sex stratified models adjusts for age, aspirin usage, metabolic syndrome status. SE, standard error; P, P-value*.

To further investigate this bias in the influence of metabolic syndrome on platelet reactivity in diabetes, we performed analyses stratifying on metabolic syndrome status and then on metabolic syndrome as well as sex. Results of the linear mixed-effect models are presented in Table 5. In the absence of metabolic syndrome, no platelet aggregation measure reached statistical significance in models combining sexes. Analyses of individuals lacking metabolic syndrome stratified by sex indicated that increased aggregation to 1 μM ADP was observed exclusively in males (β = 7.81; *P* = 0.033) and reduced aggregation to 5 μM ADP trended exclusively in females (β = 18.40; *P* = 0.058). For individuals with metabolic syndrome a similar complex pattern also emerges where the association with diabetes in response to epinephrine differed by concentration on account of sex. Increased aggregation to low concentration epinephrine was observed exclusively in males (β = 10.99; *P* = 0.007) and reduced aggregation to high concentration epinephrine exclusively in females (β = 8.50; *P* = 0.012).

**Table 5:** Results of linear mixed-effect models for association of prevalent diabetes with platelet aggregation stratified by metabolic syndrome status and by sex.

						**Males**				**Females**			
	**Phenotype**	**Beta**	**SE**	***P***	***N***	**Beta**	**SE**	***P***	***N***	**Beta**	**SE**	***P***	***N***
MS	1 μM ADP	−0.95	1.57	5.44E-01	1,218	1.47	1.90	4.40E-01	627	−3.18	2.50	2.04E-01	591
Yes	5 μM ADP	−3.70	1.48	1.22E-02	821	−3.97	2.10	5.90E-02	464	−3.60	2.02	7.45E-02	357
	0.1 μM EPI	7.32	2.99	1.42E-02	609	10.99	4.10	7.38E-03	288	3.80	4.32	3.79E-01	321
	1 μM EPI	−7.23	2.52	4.15E-03	1,003	−5.70	3.72	1.25E-01	518	−8.50	3.39	1.22E-02	485
	COLLAGEN	7.84	1.83	1.78E-05	1,297	6.20	2.44	1.11E-02	702	10.00	2.75	2.70E-04	595
MS	1 μM ADP	6.97	4.40	1.13E-01	1,780	7.81	3.67	3.32E-02	725	−18.81	16.43	2.52E-01	1,055
No	5 μM ADP	−2.95	3.69	4.23E-01	1,247	−1.15	4.42	7.94E-01	586	−18.40	9.70	5.79E-02	661
	0.1 μM EPI	4.62	9.32	6.20E-01	951	3.72	8.13	6.47E-01	321	*n*.*a*.	n.a.	n.a.	^a^630
	1 μM EPI	−7.45	7.30	3.08E-01	1,482	−4.75	8.38	5.71E-01	616	−24.38	19.52	2.12E-01	866
	COLLAGEN	7.52	4.28	7.87E-02	1,847	6.93	4.83	1.52E-01	771	8.09	14.77	5.84E-01	1,076

*Sample size (N) at different concentrations vary due to concentration titrating scheme originally applied in FHS LTA data acquisition. ADP and EPI values are % Maximal Aggregation whereas COLLAGEN is Lag time. MS, Metabolic Syndrome. Main models according to MS status adjust for age, sex, aspirin usage. Sex stratified models adjusts for age, aspirin usage*.

a *No female diabetics without metabolic syndrome were in the 0.1 μM EPI concentration testing sample. SE, standard error; P, P-value*.

Next we investigated whether platelet aggregation measures were associated with incident diabetes. During follow-up (median, 18.1 years), we observed 344 incident diabetes cases. Results of the Cox mixed-effect hazard models are presented in Table 6. After adjusting for age, sex, and aspirin usage, aggregation to 5 μM ADP was associated with a minor reduction in incident diabetes. Sex stratified analyses indicated the reduced risk was limited to females (HR = 0.974 [95%CI, 0.959–0.988] *P* = 3.3 × 10^−4^). Increased collagen lag time was also associated with a minor reduction in risk among females. These sex-specific risk reductions remained significant in multivariable models accounting for metabolic syndrome (Supplementary Table 6). We next asked whether there are sex differences in diabetes risk associated with metabolic syndrome. Remarkably, the incident diabetes hazard ratio for women with metabolic syndrome was nearly three times that for men, (HR = 14.17 [95%CI, 9.13–22.01] *P* = 2.0 × 10^−33^) vs. (HR = 5.46 [95%CI, 3.82–7.80] *P* = 2.1 × 10^−21^). Thus, we stratified the platelet aggregation analyses of incident diabetes by both sex and metabolic syndrome status. We observed the sex-specific risk reductions in incident diabetes with ADP and collagen were limited to females with metabolic syndrome (Supplementary Table 7).

**Table 6:** Results of cox mixed-effect hazard models for association of platelet aggregation with incident diabetes and stratified by sex.

**Incident diabetes**					**Males**				**Females**			
**Phenotype**	**HR**	**95% CI**	***P***	**Num**	**HR**	**95% CI**	***P***	**Num**	**HR**	**95% CI**	***P***	**Num**
1 μM ADP	0.999	0.993–1.005	0.757	310/2,763	1.003	0.993–1.013	0.528	165/1,219	0.997	0.989–1.005	0.429	145/1,544
5 μM ADP	0.990	0.981–0.999	0.021	222/1,914	0.998	0.987–1.009	0.673	132/961	0.974	0.959–0.988	3.32E-04	90/953
0.1 μM EPI	0.995	0.988–1.002	0.156	153/1,466	0.995	0.983–1.007	0.417	72/560	0.995	0.986–1.004	0.254	81/906
1 μM EPI	0.999	0.995–1.004	0.760	259/2,296	1.000	0.995–1.006	0.883	143/1,034	0.998	0.991–1.005	0.559	116/1,262
COLLAGEN	1.002	0.997–1.007	0.461	333/2,884	0.997	0.990–1.003	0.332	186/1,316	1.008	1.001–1.015	0.020	147/1,568

*Sample size (N) at different concentrations vary due to concentration titrating scheme originally applied in FHS LTA data acquisition. ADP and EPI values are % Maximal Aggregation whereas COLLAGEN is Lag time. Main model adjusts for age, sex, aspirin usage. Sex stratified models adjust for age, aspirin usage. Num indicates Events/Total at risk. HR, hazard ratio; CI, confidence interval; P, P-value*.

We broadened our investigation of platelet reactivity in incident diabetes by considering dichotomous platelet aggregation traits utilizing information available from multiple platelet agonist concentrations, as previously applied to assess risk of venous thrombosis as well as cardiovascular disease risk in the FHS cohort (32, 34). Hyperreactivity to ADP was observed at baseline in 278/3,215 (8.6%); and to epinephrine, in 314/3,086 (10.2%). Hyporeactivity to ADP was seen in 100/3,215 (3.1%); and to epinephrine, in 499/3,086 (16.2%). Results of the Cox mixed-effect hazard models for incident diabetes are presented in Supplementary Table 8. No hyper/hypo platelet reactivity traits were associated with incident diabetes.

### UK BioBank

Increased MPV is partially associated with other more established markers of increased platelet reactivity, including platelet aggregation (1). We investigated sex differences in the relationship of MPV with diabetes and medications used to manage the disease in the UK BioBank cohort. After applying exclusion criteria, the study sample consisted of 463,703 UKBB participants. Diabetes status is based on self-report of having diabetes diagnosed by a physician. Descriptive statistics for the population demographics are shown according to diabetes status in Table 7. Sex stratified descriptive statistics for platelet cell counts and MPV are presented in Supplementary Tables 9, 10, respectively. Platelet count and MPV were strongly inversely correlated (Pearson's correlation, *r* = −0.46, *P* < 2.2E−16), as is typically observed in population studies of both indices. The average participant age was 56.3 years (SD, 8.1 years), the sample was 54.8% women (*n* = 242,203), and 4.9% (*n* = 22,701) were diabetics.

**Table 7:** Baseline characteristics of UK BioBank participants.

	**Diabetic** **(*****n*** **=** **22,701)**		**Not diabetic** **(*****n*** **=** **441,002)**		
**Baseline characteristics**	**Mean (*n*)**	**SD (%)**	**Mean (*n*)**	**SD (%)**	***P*-value**
MPV, fL	9.46	1.12	9.33	1.08	6.9E-66
Age, year	59.39	7.25	56.18	8.11	<2.2E-16
Female, *n* (%)	8,483	39.5	233,720	55.6	<2.2E-16
BMI, kg/m^2^	31.28	5.9	27.17	4.61	<2.2E-16
SBP, mm Hg	143.22	18.55	139.41	19.69	1.4E-181
DBP, mm Hg	81.48	10.29	82.24	10.69	1.7E-25
Hypertension, *n* (%)	16,703	78.9	285,278	69.4	1.6E-190
BP medication, *n* (%)	14,060	61.9	77,802	17.6	<2.2E-16
Hyperlipidemia medication, *n* (%)	16,646	73.3	59,900	13.6	<2.2E-16
Anti-platelet medication, *n* (%)	11,779	18.7	10,922	2.7	<2.2E-16
Diabetes medication, *n* (%)	15,847	69.8			
Metformin, *n* (%)	12,475	54.9			
Insulin, *n* (%)	4,336	19.1			
Sulphonylurea, *n* (%)	4,723	20.8			

*BMI indicates body mass index; HDL, high-density lipoprotein; LDL, low-density lipoprotein; SBP, systolic blood pressure; DBP, diastolic blood pressure; BP, blood pressure; n, sample size; SD, standard deviation; P-value, univariate T-test P-value. Medication usage is based on self-report*.

We first investigated the association between diabetes and MPV. Results of the multivariable linear model are presented in Table 8. Diabetes was associated with increased MPV (β = 0.0976; *P* = 8.62 × 10^−33^) after adjusting for age, sex, BMI, hypertension, as well as anti-platelet and cholesterol medication. We observed a strong interaction between sex and diabetes status (*P* = 3.17 × 10^−11^). Stratifying the analysis of diabetics by sex, we observed the association with increased MPV is markedly stronger in males (β = 0.1232; *P* = 1.00 × 10^−31^) than females (β = 0.0514; *P* = 7.37 × 10^−5^).

**Table 8:** Results of multivariable linear model for association of self-reported diabetes with MPV and stratified by sex.

	**All (*****n*** **=** **440,393)**			**Males (*****n*** **=** **198,969)**			**Females (*****n*** **=** **241,424)**			
**Phenotype**	**Beta**	**SE**	***P***	**Beta**	**SE**	***P***	**Beta**	**SE**	***P***	***P*_**inter**_**
MPV	0.0976	0.0082	8.62E-33	0.1232	0.0105	1.00E-31	0.0514	0.0130	7.37E-05	3.17E-11

*Main model adjusts for age, sex, BMI, hypertension, anti-platelet, and cholesterol medication usage. Stratified models adjust for age, BMI, hypertension, anti-platelet and cholesterol medication usage. Medication usage is based on self-report. SE, standard error; P, P-value; P_inter_, P-value for interaction between diabetes status and sex*.

In order to obtain interpretable risk estimates based on MPV, we subsequently ran multivariable logistic model associations of MPV with diabetes. MPV was associated with an increase in prevalent diabetes (OR = 1.08 [95%CI, 1.07–1.10] *P* = 3.3 × 10^−4^) after adjusting for age, sex, BMI, hypertension, as well as anti-platelet and cholesterol medication. A significant interaction with MPV was found for sex (*P* = 3.17 × 10^−11^) (Table 9). As demonstrated in sex-stratified analyses, the magnitude of the association was greater for men (OR = 1.11 [95%CI, 1.09–1.13] *P* = 3.3 × 10^−4^) than women (OR = 1.05 [95%CI, 1.02–1.07] *P* = 4.3 × 10^−5^).

**Table 9:** Results of multivariable logistic regression model for association of MPV with self-reported diabetes and stratified by sex.

	**All (*****n*** **=** **440,393)**			**Males (*****n*** **=** **198,969)**			**Females (*****n*** **=** **241,424)**			
**Phenotype**	**OR**	**95% CI**	***P***	**OR**	**95% CI**	***P***	**OR**	**95% CI**	***P***	***P*_**inter**_**
MPV	1.08	1.07–1.10	6.49E-30	1.11	1.09–1.13	3.16E-30	1.05	1.02–1.07	4.28E-05	6.80E-05

*Main model adjusts for age, sex, BMI, hypertension, anti-platelet, and cholesterol medication usage. Stratified models adjust for age, BMI, hypertension, anti-platelet, and cholesterol medication usage. Medication usage is based on self-report. OR indicates odds ratio; CI, confidence interval; P, P-value; P_inter_, P-value for interaction between MPV and sex*.

We next investigated the relationship between diabetic drug therapies and MPV. More than two-thirds (69.8%, *n* = 15,847) reported taking medication to manage their diabetes, the most common were Metformin (54.9%, *n* = 12,475), Insulin (19.1%, *n* = 4,336), and the sulphonylureas (20.8%, *n* = 4,723). Results of the multivariable linear models adjusting for age, sex, BMI, hypertension, as well as anti-platelet and cholesterol medication are presented in Table 10. Increased MPV was associated with Insulin (β = 0.1341; *P* = 1.38 × 10^−11^) whereas decreased MPV was associated with both Metformin (β = 0.0763; *P* = 1.99 × 10^−6^) as well as sulphonylureas (β = 0.0559; *P* = 0.0034). Drug-sex interactions were significant for Metformin and Insulin, but not sulphonylureas. In sex-stratified analyses each drug showed the same direction of effect in both sexes, however, the magnitude of the association with MPV was nearly twice as great or more in females compared to males (Table 10). We investigated whether there were sex differences in diabetes medication rates that could be impacting the associations we observed with MPV. If a bias were to follow the trend from the association of MPV with diabetes (Table 9), we reasoned Insulin-dependent diabetes rates may be much higher in male cases whereas females may more often be taking Metformin and sulphonylureas. We did not observe this pattern in medication usage. The difference in Insulin-dependent diabetes rates between males (18.9%, *n* = 2,580) and females (19.4%, *n* = 1,756) was not statistically significant (χ^2^ test, *P* = 0.32). A greater proportion of males (56.9%, *n* = 7,772) than females (52.0%, *n* = 4,703) reported taking Metformin (χ2 *test, P* = 5.92 × 10^−13^). Finally, a greater proportion of males (23.4%, *n* = 3,192) than females (16.9%, *n* = 1,531) reported taking sulphonylureas (χ2 *test, P* = 1.88 × 10^−31^).

**Table 10:** Results of multivariable linear models for association of diabetes medication usage with MPV and stratified by sex.

	**All diabetics (*****n*** **=** **21,314)**			**Male (*****n*** **=** **12,896)**			**Female (*****n*** **=** **8,418)**			
**Medication**	**Beta**	**SE**	***P***	**Beta**	**SE**	***P***	**Beta**	**SE**	***P***	***P*_**inter**_**
Insulin	0.1341	0.0198	1.38E-11	0.1058	0.0257	3.75E-05	0.1782	0.0313	1.27E-08	2.81E-06
Metformin	−0.0763	0.0160	1.99E-06	−0.0504	0.0206	1.44E-02	−0.1205	0.0257	2.75E-06	6.42E-06
Sulphonylureas	−0.0559	0.0191	3.41E-03	−0.0391	0.0236	9.71E-02	−0.0888	0.0327	6.64E-03	0.077

*Main model adjusts for age, sex, BMI, hypertension, anti-platelet, and cholesterol medication usage. Stratified models adjust for age, BMI, hypertension, anti-platelet, and cholesterol medication usage. Medication usage is based on self-report. SE, standard error; P, P-value; P_inter_, P-value for interaction between medication and sex*.

## Discussion

We do not observe a clear and consistent pattern of association between ADP, collagen, or epinephrine measures of platelet function by LTA with metabolic syndrome or diabetes. This is consistent with what we observed prior for VTE (34), but contrasts with arterial disease where there was a strong association of ADP-driven hyper-reactivity with future thrombosis (32). Despite the large body of research concerning the role of platelets in diabetes, evidence from population-based studies of platelet aggregation in diabetes is limited. Study samples are often selected on the basis of comorbidities including CAD (13, 38) or disease severity, such as patients hospitalized for inadequate glycemic control (20). Our study results did not change appreciably if we adjusted for, or stratified, diabetes associations on aspirin use, or on CVD status. Many clinical studies were limited to one sex (10, 15) and the majority have not accounted for sex as a confounding factor. While women in general possess greater platelet reactivity (30), our results do suggest that platelet reactivity associated with both metabolic syndrome and diabetes is reduced in women compared to men. Recently, there was a study of 81 diabetic men and 533 non-diabetic men with platelet aggregation assays of ADP, AA, collagen, thrombin receptor activating peptide (TRAP) and protease-activated receptor 4 agonist peptide (PAR-4AP) that also reported negative findings in regards to platelet function measurements and diabetes status, whether in aspirin or non-aspirin takers (39).

MPV has been suggested as a potential surrogate metric for other established markers of platelet activity including platelet aggregation, though evidence has been mixed (22). Since platelet aggregation has only been measured in cohorts of several thousands and often in clinical centers primarily focused on platelet and bleeding disorders, utilizing MPV in research measurements has the potential to access much larger populations and clinical cohort samples. Utilizing data from the UK BioBank we conducted the largest multivariable analysis of MPV and diabetes to date. While the effect size is modest, MPV appears to be robustly associated with diabetes and differs in magnitude according to sex. The effect among females was less than half of that observed in males. In contrast, among non-diabetics MPV is increased in females compared to males (Supplementary Table 10) an observation consistent with sex differences observed in platelet aggregation studies (30). The ability to detect this association with sex in the general population may depend on the sample size. Increased MPV in females is observed in reference measurements of healthy populations including more than 12,000 subjects (40, 41), but not fewer (42, 43).

We observed strong associations between MPV and several medications used to manage diabetes, including insulin, metformin, and sulphonylureas. In each case, there is evidence for a biological basis to the association. This is the first study to show an association between insulin therapy and MPV in diabetics. Two previous studies (with fewer than 350 insulin-dependent diabetics in each) did not (23, 27). Insulin has been shown to reduce platelet aggregation in healthy subjects both *in vitro* and *in vivo* (44). Insulin inhibits Ca^2+^ mobilization in platelets by interfering with P2Y_12_-mediated cAMP suppression (45). The platelets of diabetics have decreased sensitivity to insulin, potentially leading to reduced P2Y_12_ inhibition and increased platelet reactivity (46). The strong increase in MPV associated with insulin therapy may be explained by the fact that patients with insulin-treated diabetes are likely to be at a more advanced stage of insulin resistance (47). This may further explain increased MPV in females because women are more intrinsically insulin resistant than men (48).

Metformin has been shown to reduce platelet aggregation to ADP in insulin-dependent diabetics (49). This may be related to the drug's protective effect on mitochondrial function. Hyperglycemia induces mitochondrial hyperpolarization in platelets, resulting in increased reactive oxygen species (ROS) generation and subsequent activation (50). Metformin inhibits mitochondrial complex I and thereby reduces activated platelet-induced mitochondrial hyperpolarization, ROS overload, and inhibits mitochondrial DNA release (51). Metformin therapy reduces plasma levels of interleukin-6 (IL-6), an inflammatory cytokine associated with insulin resistance and diabetes risk (52). IL-6 functions in a paracrine signaling loop, stimulating thrombopoietin (TPO) in the liver which increases platelet production in the bone marrow (53). Metformin inhibits hepatic IL-6 signaling *in vivo* (54). While metformin might act to inhibit IL-6/TPO and platelet generation, it is unclear to us how this might lead to a concomitant decrease in the average size of platelets, given that PLT and MPV are typically strongly inversely correlated. However, one indication that there could be a causal effect of metformin on reducing platelet volumes comes from a study of 60 newly diagnosed diabetics treated with metformin monotherapy. After 6 months from baseline, the metformin-treated individuals had a significant reduction in MPV (55).

Inhibition of platelet aggregation by sulphonylureas has also been demonstrated *in vitro* (56, 57). However, a clinical trial of Gliclazide failed to show an effect on platelet aggregation in either insulin- or non-insulin-treated diabetics (58). For the most commonly prescribed sulphonylurea, Gliclazide, the mechanism may involve inhibition of activated glycogen synthetase, activation of adenylate cyclase, modulation of arachidonic acid release from platelet membranes, stimulation of prostacyclin production, and inhibition of the pro-aggregating action of thromboxane A2 (59). Gliclazide therapy has also been shown to reduce IL-6 levels in diabetics presenting with poor glucose control (60), and similar to metformin we speculate that changes in the IL-6/TPO axis could mediate feedback effects on platelet generation, turnover and the average size of platelets.

Sex differences with respect to platelet reactivity were apparent in the FHS as well as UK BioBank cohorts. In the former, platelet aggregation associated with both metabolic syndrome and diabetes was reduced in women compared to men. In the latter, the increase in MPV associated with diabetes among women was less than half of that observed in men. Further, the association of diabetes medications with MPV was nearly twice as great or more in women compared to men. Women are distinctly different from men with regard to the actions of insulin and the susceptibility to develop insulin resistance (48). Our results indicate it will be important for future studies to consider sex differences and medication status. Diabetes, even at an early stage, attenuates the protective effect of female sex on CVD and increases the risk for CVD in females to a greater extent than in males (61). While the effect size of MPV may be small it may prove a useful variable in prediction models for diabetes, especially given the importance of sex differences in its biological risk factors and pathophysiological mechanisms. The precise mechanisms whereby diabetes status influences MPV remains to be studied further and may include complex interactions between disease progression, sex, medications, genetic alleles influencing MPV or diabetes, and effects on platelet generation and turnover.

### Limitations

Though LTA is the gold standard, it is still an *in vitro* rather *in vivo* measure of platelet activation. Although, LTA in FHS was performed by lab staff experienced with a high volume of samples, LTA can be subject to technical variability including operator influences that can potentially harm accuracy or generalizability of results (62). We also were limited to those historically measured agonists and concentrations available within FHS. Other pathways of platelet activation (e.g., thrombin) or approaches to platelet function (e.g., whole blood assays, granule release markers, other platelet morphology assays like spreading and imaging-based assays, shear stress flow systems) were not available for analysis in the FHS Offspring cohort at Exam 5. While all regression models accounted for aspirin usage, this was determined by an indirect measure (failure to demonstrate full aggregation response to arachidonic acid). Whether and when participants precisely took aspirin could not be determined. However, a recent study found that arachidonic acid LTA provided the best discrimination of aspirin use in diabetics among nine platelet function tests examined (63). Both populations we examined are of predominantly European ancestry which could limit the generalizability of the results. Some prior work such as the large NHANES study (*n* = 17,969) suggest that African or Hispanic populations may have higher MPV than European ancestry populations, though the absolute mean difference in NHANES was small (<0.17 femptoliters) (64). There are some limitations unique to the UK BioBank analyses. First, diabetes status is based on self-report, though that of being diagnosed by a physician. We were unable to consider direct measures of several lipid (HDL, triglycerides) and nonlipid (fasting blood glucose) risk factors which would have allowed us to classify participants with metabolic syndrome. However, our models did adjust for BMI, hypertension and indirect evidence of lipid and thrombotic risk, cholesterol lowering and antiplatelet medications, respectively. MPV measurements seem to be effected by pre-analytic factors and analytic factors like timing, temperature, tube type, and anticoagulant, and instrument more so than most other cell count measurements (65–67). A strength of this study is that a single tube type and instrument model were used to measure MPV in a large sample, though this could also limit generalizability to other studies with different instruments or tubes. Due to the size and nature of the study UK BioBank samples were shipped overnight and stored at 4°C. We cannot determine whether these conditions may have impacted diabetes-MPV associations. However, we do not expect storage and transport time to be specifically related to diabetes status, thus, we expect there would be little confounding of our associations. The associations of MPV with Metformin, Insulin, and sulphonylureas are also based on self-report. Retrospective analyses of in the UK have estimated medication adherence to oral hypoglycemics to be 81.6% for monotherapy and 80.8% for dual therapy (68). Adherence to insulin therapy in the UK is estimated to be 70.6% (69).

In conclusion, platelet function as measured by aggregation does not appear to be consistently associated with diabetes. In agreement with prior studies MPV is robustly associated suggesting future work may focus on how MPV segments pre-diabetics and diabetics for risk prediction as well as how this may differ by treatment and sex.

## Data Availability Statement

The datasets generated for this study will not be made publicly available. Data from The Framingham Heart Study is available through application on dbGaP (https://www.ncbi.nlm.nih.gov/projects/gap/cgi-bin/study.cgi?study_id=phs000007.v30.p11). Data from UK BioBank is available through application to UK BioBank (https://www.ukbiobank.ac.uk/researchers/).

## Ethics Statement

The studies involving human participants were reviewed and approved by Boston University Institutional Review Board. The patients/participants provided their written informed consent to participate in this study.

## Author Contributions

BR conducted all statistical analyses, and BR and AJ wrote the manuscript. Both authors critically edited the manuscript and approved the final submission.

## Conflict of Interest

The authors declare that the research was conducted in the absence of any commercial or financial relationships that could be construed as a potential conflict of interest.
