# Supplementary material for: Platelet Measurements and Type 2 Diabetes: Investigations in Two Population-Based Cohorts
Source: Front Cardiovasc Med. 2020 Jul 10;7:118. doi: 10.3389/fcvm.2020.00118 (PMC7365849; doi:10.3389/fcvm.2020.00118)
Supplement: Supplementary file 1 [file Data_Sheet_1.docx]

**Supplementary Figure 1, Distributions of responses across different ADP concentrations**


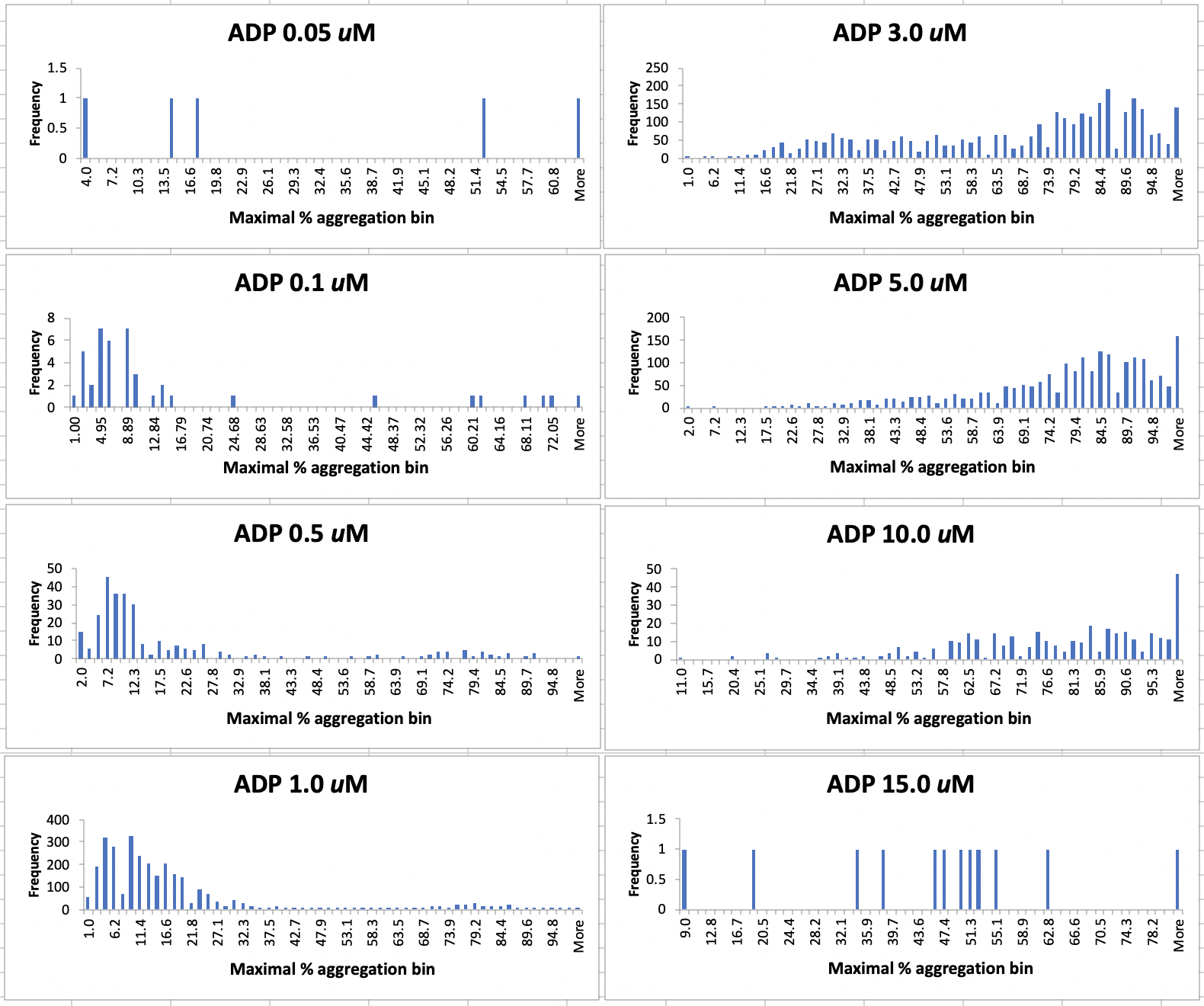


**Supplementary Figure 2, Distributions of epinephrine responses across different epinephrine concentrations**


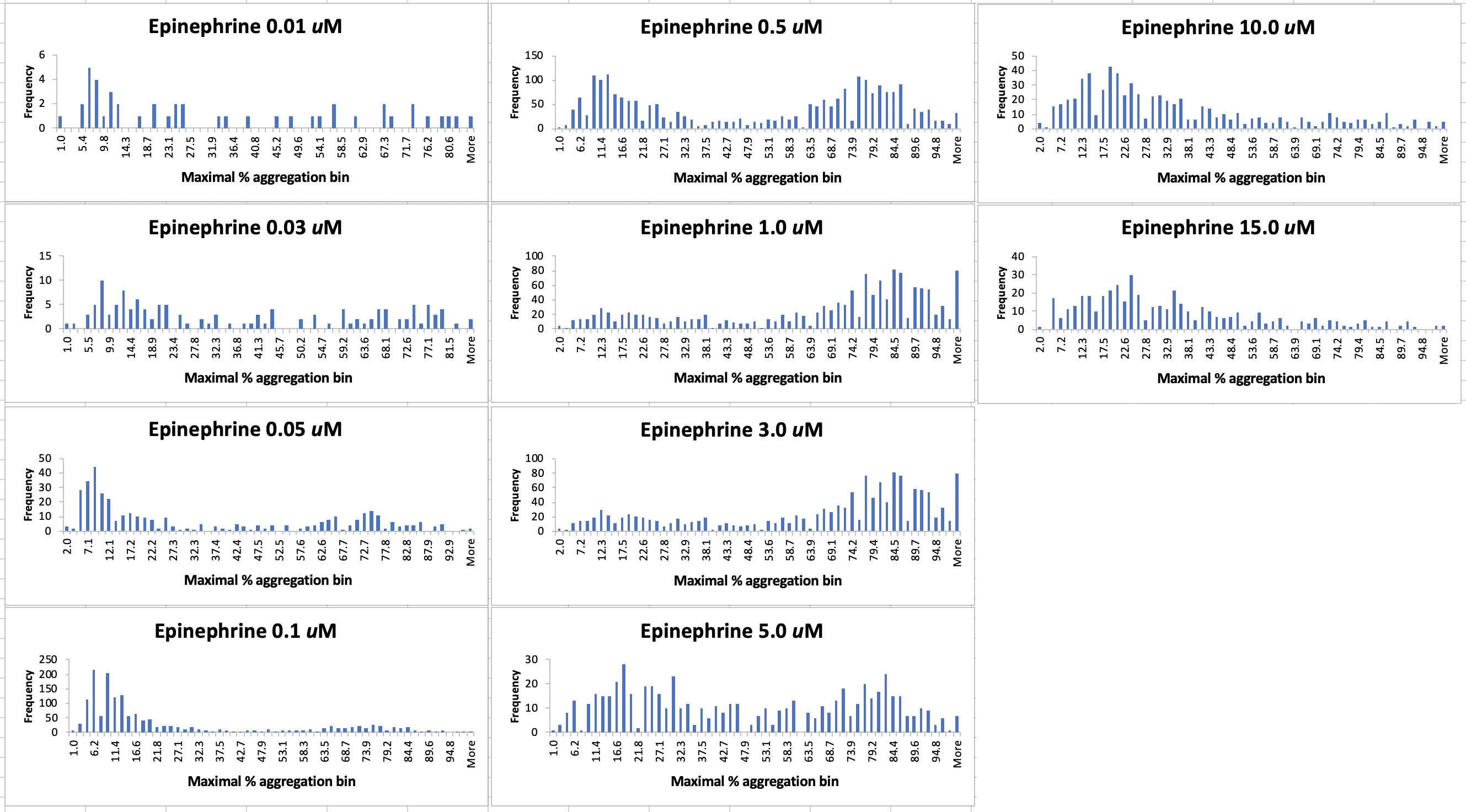


**Supplementary Table 1, Results of Linear Mixed-Effect Models for Association Seperately of either Age or Sex with Platelet Aggregation**

|  | Female Sex | | | | Age | | | |
| --- | --- | --- | --- | --- | --- | --- | --- | --- |
| Phenotype | Beta | SE | P | N | Beta | SE | P | N |
| 1 µM ADP | **7.07** | **0.74** | **1.21E-21** | **2998** | **0.22** | **0.04** | **1.57E-08** | **2998** |
| 5 µM ADP | **3.40** | **0.67** | **3.67E-07** | **2068** | **0.11** | **0.04** | **0.0019** | **2068** |
| 0.1 µM EPI | **5.89** | **1.31** | **7.34E-06** | **1560** | **0.19** | **0.07** | **0.0048** | **1560** |
| 1 µM EPI | **6.20** | **1.15** | **7.62E-08** | **2485** | **0.13** | **0.06** | **0.035** | **2485** |
| COLLAGEN | **-5.11** | **0.80** | **1.99E-10** | **3144** | 0.01 | 0.04 | 0.79 | 3144 |

| *sample size at different concentrations vary do to concentration titrating scheme originally applied in FHS LTA data acquisition | | | |
| --- | --- | --- | --- |
| ADP and EPI values are % Maximal Aggregation whereas COLLAGEN is Lag time. |  |  |  |
| SE, standard error; P, P value; N, sample size. | | |  |
|  | |  |  |

**Supplementary Table 2, Results of Linear Mixed-Effect Models for Association of Metabolic Syndrome with Platelet Aggregation Excluding Current Diabetics and Stratified by Sex**

| Phenotype | Covariate | | Beta | | SE | | P | | N | | Beta | SE | | P | | N | | Beta | SE | | P | | N | |  |  |  |
| --- | --- | --- | --- | --- | --- | --- | --- | --- | --- | --- | --- | --- | --- | --- | --- | --- | --- | --- | --- | --- | --- | --- | --- | --- | --- | --- | --- |
| 1 µM ADP | MS | | 0.27 | | 0.82 | | 7.39E-01 | | 2783 | | 1.12 | 0.96 | | 2.46E-01 | | 1227 | | -0.75 | 1.29 | | 5.60E-01 | | 1556 | |  |  |  |
| 5 µM ADP | MS | | **-2.57** | | **0.75** | | **5.68E-04** | | **1925** | | **-2.22** | **1.10** | | **4.35E-02** | | **965** | | **-3.28** | **0.99** | | **8.85E-04** | | **960** | |  |  |  |
| 0.1 µM EPI | MS | | **-3.46** | | **1.41** | | **1.42E-02** | | **1480** | | -1.10 | 1.92 | | 5.67E-01 | | 565 | | **-5.36** | **1.97** | | **6.42E-03** | | **915** | |  |  |  |
| 1 µM EPI | MS | | **-2.17** | | **1.29** | | **9.17E-02** | | **2315** | | -1.03 | 1.93 | | 5.93E-01 | | 1042 | | **-3.50** | **1.72** | | **4.23E-02** | | **1273** | |  |  |  |
| COLLAGEN | MS | | 1.03 | | 0.88 | | 2.44E-01 | | 2903 | | 0.33 | 1.30 | | 8.02E-01 | | 1324 | | **2.02** | **1.21** | | **9.35E-02** | | **1579** | |  |  |  |
|  | |  | |  | |  | |  | |  | | |  | |  | |  | | |  | |  | |  | |  |  |
| *sample size at different concentrations vary do to concentration titrating scheme originally applied in FHS LTA data acquisition | | | | | | | | | | | | | | | | | | | | | |  | |  | |  |  |
| ADP and EPI values are % Maximal Aggregation whereas COLLAGEN is Lag time. | | | | | | | | | | | | | | |  | |  | | |  | |  | |  | |  |  |
| Main model adjusts for age, sex, aspirin usage. Sex stratified model adjusts for age, aspirin usage. | | | | | | | | | | | | | | | | | | | |  | |  | |  | |  |  |
| MS indicates Metabolic Syndrome; SE, standard error; P, P value; N, sample size. | | | | | | | | | | | | | | | | |  | | |  | |  | |  | |  |  |

**Supplementary Table 3, Results of Linear Mixed-Effect Models for Association of Sex with Platelet Aggregation in Individuals Free of Metabolic Syndrome and Diabetes**

| Phenotype | Covariate | Beta (relative to female sex) | SE | P | N |
| --- | --- | --- | --- | --- | --- |
| 1 µM ADP | SEX | **8.26** | **1.03** | **1.22E-15** | **1686** |
| 5 µM ADP | SEX | **3.79** | **0.90** | **2.40E-05** | **1175** |
| 0.1 µM EPI | SEX | **7.15** | **1.86** | **1.21E-04** | **901** |
| 1 µM EPI | SEX | **7.01** | **1.56** | **7.26E-06** | **1401** |
| COLLAGEN | SEX | **-5.56** | **1.03** | **7.02E-08** | **1743** |
|  |  |  |  |  |  |
| *sample size at different concentrations vary do to concentration titrating scheme originally applied in FHS LTA data acquisition | | | | | |
| ADP and EPI values are % Maximal Aggregation whereas COLLAGEN is Lag time. | | | | | |
| Model adjusts for age, and aspirin usage. | | |  |  |  |
| SE, standard error; P, P value; N, sample size. | | | |  |  |

**Supplementary Table 4**. Results of Linear Mixed-Effect Models for Association of Prevalent Diabetes with Platelet Aggregation Stratified by Sex (or not), and without Aspirin adjustment or stratified by Aspirin Status.

|  |  |  |  |  |  | Male | | | | Female | | | |
| --- | --- | --- | --- | --- | --- | --- | --- | --- | --- | --- | --- | --- | --- |
| Phenotype | Analysis | Beta | SE | P | N | Beta | SE | P | N | Beta | SE | P | N |
| 1 uM ADP | No aspirin adjustment | -0.61 | 1.46 | 0.68 | 2998 | 1.89 | 1.60 | 0.24 | 1352 | -4.25 | 2.50 | 0.09 | 1646 |
| 1 uM ADP | Excluding aspirin takers | -0.28 | 1.78 | 0.87 | 2587 | 3.54 | 2.08 | 0.09 | 1111 | -4.88 | 2.82 | 0.08 | 1476 |
| 1 uM ADP | Including only aspirin takers | 0.82 | 1.14 | 0.47 | 411 | 0.92 | 1.28 | 0.47 | 241 | 1.26 | 2.31 | 0.59 | 170 |
| 5 uM ADP | No aspirin adjustment | **-7.37** | **1.60** | **3.90E-06** | 2068 | **-8.00** | **2.29** | **4.66E-04** | **1050** | **-7.62** | **2.20** | **5.33E-04** | **1018** |
| 5 uM ADP | Excluding aspirin takers | **-5.19** | **1.58** | **1.01E-03** | 1737 | -4.11 | 2.37 | 0.08 | 846 | **-6.51** | **2.06** | **1.59E-03** | **891** |
| 5 uM ADP | Including only aspirin takers | -2.53 | 2.62 | 0.33 | 331 | -3.50 | 3.11 | 0.26 | 204 | -1.28 | 4.72 | 0.79 | 127 |
| 0.1 uM Epi | No aspirin adjustment | 4.80 | 2.92 | 0.10 | 1560 | **9.22** | **3.59** | **0.01** | **609** | -0.20 | 4.53 | 0.97 | 951 |
| 0.1 uM Epi | Excluding aspirin takers | 5.38 | 2.98 | 0.07 | 1551 | **9.97** | **3.68** | **0.01** | **603** | 0.26 | 4.60 | 0.95 | 948 |
| 0.1 uM Epi | Including only aspirin takers | 2.98 | 1.61 | 0.06 | 9 | 3.24 | 8.95 | 0.72 | 6 | n.a. | n.a. | n.a. | 3 |
| 1 uM Epi | No aspirin adjustment | **10.75** | **2.47** | **1.39E-05** | **2485** | **-9.97** | **3.56** | **0.01** | **1134** | **-11.92** | **3.44** | **5.23E-04** | **1351** |
| 1 uM Epi | Excluding aspirin takers | **-8.82** | **2.57** | **6.09E-04** | **2324** | -6.57 | 3.84 | 0.09 | 1040 | **-11.40** | **3.44** | **9.13E-04** | **1284** |
| 1 uM Epi | Including only aspirin takers | -2.44 | 1.86 | 0.19 | 161 | -1.19 | 2.01 | 0.55 | 94 | -5.92 | 3.92 | 0.13 | 67 |
| COLLAGEN | No aspirin adjustment | **10.91** | **1.65** | **4.16E-11** | **3144** | **10.50** | **2.26** | **3.39E-06** | **1473** | **12.28** | **2.49** | **8.46E-07** | **1671** |
| COLLAGEN | Excluding aspirin takers | **4.98** | **1.63** | **2.19E-03** | **2659** | 4.02 | 2.22 | 0.07 | 1173 | **7.12** | **2.41** | **3.08E-03** | **1486** |
| COLLAGEN | Including only aspirin takers | **16.27** | **4.26** | **1.33E-04** | **485** | **11.46** | **5.05** | **0.02** | **300** | **28.38** | **8.11** | **4.68E-04** | **185** |

| *sample size at different concentrations vary do to concentration titrating scheme originally applied in FHS LTA data acquisition | | | |
| --- | --- | --- | --- |
| ADP and EPI values are % Maximal Aggregation whereas COLLAGEN is Lag time. | | | |
| Model adjusts for age (and sex in combined model). |  |  |  |
| SE, standard error; P, P value; N, sample size. | |  |  |

**Supplementary Table 5**. Results of Linear Mixed-Effect Models for Association of Prevalent Diabetes with Platelet Aggregation Stratified by Sex (or not), and without Prior CVD Adjustment or Stratified by Prior CVD Status.

|  |  |  |  |  |  | Male | | | | Female | | | |
| --- | --- | --- | --- | --- | --- | --- | --- | --- | --- | --- | --- | --- | --- |
| Phenotype | Analysis | Beta | SE | P | N | Beta | SE | P | N | Beta | SE | P | N |
| 1 uM ADP | Adjusting for prior CVD | 0.038 | 1.46 | 0.979191 | 2998 | 2.5859 | 1.6 | 0.1061 | 1352 | -3.952 | 2.5 | 0.11325 | 1646 |
| 1 uM ADP | Including only individuals with prior CVD | -0.3 | 2.63 | 0.909522 | 279 | 2.682 | 2.828 | 0.343 | 175 | -5.509 | 5 | 0.271176 | 104 |
| 1 uM ADP | Excluding individuals with prior CVD | 0.313 | 1.67 | 0.850749 | 2719 | 2.7232 | 1.866 | 0.1444 | 1177 | -3.677 | 2.7 | 0.180845 | 1542 |
| 5 uM ADP | Adjusting for prior CVD | **-4.48** | **1.35** | **0.00095** | **2068** | **-3.8** | **1.927** | **0.0486** | **1050** | **-5.64** | **1.9** | **0.002768** | **1018** |
| 5 uM ADP | Including only individuals with prior CVD | **-7.58** | **3** | **0.011591** | **177** | -4.95 | 3.722 | 0.1836 | 116 | **-15.17** | **4.7** | **0.001296** | **61** |
| 5 uM ADP | Excluding individuals with prior CVD | **-3.74** | **1.51** | **0.013097** | **1891** | -3.394 | 2.209 | 0.1245 | 934 | **-4.09** | **2** | **0.043105** | **957** |
| 0.1 uM Epi | Adjusting for prior CVD | 5.126 | 2.94 | 0.080922 | 1560 | **9.9238** | **3.603** | **0.0059** | **609** | -0.084 | 4.5 | 0.985294 | 951 |
| 0.1 uM Epi | Including only individuals with prior CVD | 6.509 | 7.34 | 0.375197 | 105 | 5.4297 | 8.628 | 0.5292 | 59 | 4.872 | 13 | 0.709593 | 46 |
| 0.1 uM Epi | Excluding individuals with prior CVD | 5.113 | 3.21 | 0.111303 | 1455 | **10.569** | **3.973** | **0.0078** | **550** | -1.05 | 4.9 | 0.829673 | 905 |
| 1 uM Epi | Adjusting for prior CVD | **-7.83** | **2.33** | **0.000795** | **2485** | -5.57 | 3.358 | 0.0972 | 1134 | **-10.38** | **3.2** | **0.001397** | **1351** |
| 1 uM Epi | Including only individuals with prior CVD | -1.88 | 5.14 | 0.714674 | 184 | 0.8762 | 6.957 | 0.8998 | 105 | -3.808 | 7.6 | 0.614451 | 79 |
| 1 uM Epi | Excluding individuals with prior CVD | **-9.03** | **2.57** | **4.44E-04** | **2301** | -7.165 | 3.726 | 0.0545 | 1029 | **-11.18** | **3.6** | **1.67E-03** | **1272** |
| COLLAGEN | Adjusting for prior CVD | **8.063** | **1.55** | **1.81E-07** | **3144** | **6.3733** | **2.086** | **0.0023** | **1473** | **10.98** | **2.3** | **2.92E-06** | **1671** |
| COLLAGEN | Including only individuals with prior CVD | 5.607 | 3.55 | 0.114114 | 297 | 4.8279 | 4.561 | 0.2898 | 192 | 8.408 | 5.4 | 0.119975 | 105 |
| COLLAGEN | Excluding individuals with prior CVD | **8.316** | **1.72** | **1.35E-06** | **2847** | **6.6197** | **2.349** | **0.0048** | **1281** | **11.42** | **2.6** | **8.76E-06** | **1566** |

| *sample size at different concentrations vary do to concentration titrating scheme originally applied in FHS LTA data acquisition | | | |
| --- | --- | --- | --- |
| ADP and EPI values are % Maximal Aggregation whereas COLLAGEN is Lag time. | | | |
| Model adjusts for age (and sex in combined model). |  |  |  |
| SE, standard error; P, P value; N, sample size.  CVD was defined as previously analyzed in Puurunen et al., PubMed ID 29502103, and included MI, angina pectoris, stroke, transient ischemic attack, coronary insufficiency, atherothrombotic infraction and congestive heart failure. | |  |  |

**Supplementary Table 6, Results of Cox Mixed-Effect Hazard Models for Association of Platelet Aggregation with Incident Diabetes Accounting for Metabolic Syndrome and Stratified by Sex**

|  |  |  |  |  |  | Male | | | | | Female | | | | |
| --- | --- | --- | --- | --- | --- | --- | --- | --- | --- | --- | --- | --- | --- | --- | --- |
| Phenotype | HR | CI95 low | CI95 high | P | N | HR | CI95 low | CI95 high | P | N | HR | CI95 low | CI95 high | P | N |
| 1 µM ADP | 0.999 | 0.993 | 1.005 | 7.43E-01 | 310;2763 | 1.001 | 0.990 | 1.011 | 9.14E-01 | 165;1219 | 0.998 | 0.991 | 1.006 | 6.86E-01 | 145;1544 |
| 5 µM ADP | 0.995 | 0.985 | 1.005 | 3.00E-01 | 222;1914 | 1.001 | 0.989 | 1.013 | 8.67E-01 | 132;961 | **0.984** | **0.968** | **1.000** | **4.90E-02** | **90;953** |
| 0.1 µM EPI | 0.997 | 0.989 | 1.004 | 3.61E-01 | 153;1466 | 0.994 | 0.982 | 1.007 | 3.41E-01 | 72;560 | 0.999 | 0.989 | 1.008 | 7.68E-01 | 81;906 |
| 1 µM EPI | 1.001 | 0.996 | 1.006 | 6.60E-01 | 259;2296 | 1.001 | 0.995 | 1.007 | 8.06E-01 | 143;1034 | 1.002 | 0.995 | 1.010 | 5.47E-01 | 116;1262 |
| COLLAGEN | 1.001 | 0.996 | 1.006 | 6.92E-01 | 333;2884 | 0.996 | 0.990 | 1.003 | 2.69E-01 | 186;1316 | **1.007** | **1.000** | **1.013** | **4.90E-02** | **147;1568** |
|  |  |  |  |  |  |  |  |  |  |  |  |  |  |  |  |
| *sample size at different concentrations vary do to concentration titrating scheme originally applied in FHS LTA data acquisition | | | | | | | | | | |  |  |  |  |  |
| ADP and EPI values are % Maximal Aggregation whereas COLLAGEN is Lag time. | | | | | | | |  |  |  |  |  |  |  |  |
| Main model adjusts for age, sex, metabolic syndrome status, and aspirin usage. Sex stratified model adjusts for age, metabolic syndrome status, and aspirin usage. | | | | | | | | | | | | | | | |
| HR indicates Hazard ratio; CI95, 95% confidence intervals; P, P value; N, number of events;total at risk. | | | | | | | | | |  |  |  |  |  |  |

**Supplementary Table 7, Results of Cox Mixed-Effect Hazard Models for Association of Platelet Aggregation with Incident Diabetes Stratified by Metabolic Syndrome Status and Sex**

| MS Yes | All | | | | | Male | | | | | Female | | | | |
| --- | --- | --- | --- | --- | --- | --- | --- | --- | --- | --- | --- | --- | --- | --- | --- |
| Model | HR | CI95 low | CI95 high | P | N | HR | CI95 low | CI95 high | P | N | HR | CI95 low | CI95 high | P | N |
| 1 µM ADP | 1.000 | 0.993 | 1.006 | 9.26E-01 | 245;1012 | 1.001 | 0.990 | 1.012 | 8.27E-01 | 126;517 | 0.999 | 0.990 | 1.007 | 7.60E-01 | 119;495 |
| 5 µM ADP | 0.994 | 0.983 | 1.005 | 2.80E-01 | 172;689 | 1.002 | 0.988 | 1.016 | 7.70E-01 | 96;393 | **0.979** | **0.961** | **0.998** | **2.73E-02** | **76;296** |
| 0.1 µM EPI | 0.999 | 0.991 | 1.008 | 8.55E-01 | 115;527 | 0.995 | 0.981 | 1.009 | 4.41E-01 | 55;249 | 1.002 | 0.992 | 1.013 | 6.56E-01 | 60;278 |
| 1 µM EPI | 1.000 | 0.995 | 1.005 | 9.96E-01 | 200;836 | 1.001 | 0.994 | 1.008 | 7.64E-01 | 107;435 | 0.999 | 0.991 | 1.007 | 7.74E-01 | 93;401 |
| COLLAGEN | 1.003 | 0.997 | 1.008 | 3.16E-01 | 262;1070 | 0.997 | 0.989 | 1.004 | 3.60E-01 | 142;572 | **1.009** | **1.002** | **1.015** | **9.79E-03** | **120;498** |
|  |  |  |  |  |  |  |  |  |  |  |  |  |  |  |  |
| MS No | All | | | | | Male | | | | | Female | | | | |
| Model | HR | CI95 low | CI95 high | P | N | HR | CI95 low | CI95 high | P | N | HR | CI95 low | CI95 high | P | N |
| 1 µM ADP | 0.996 | 0.981 | 1.012 | 6.33E-01 | 65;1751 | 0.996 | 0.969 | 1.023 | 7.49E-01 | 39;702 | 0.996 | 0.979 | 1.014 | 6.88E-01 | 26;1049 |
| 5 µM ADP | 1.000 | 0.982 | 1.018 | 9.61E-01 | 50;1225 | 0.999 | 0.980 | 1.019 | 9.40E-01 | 36;568 | 1.003 | 0.960 | 1.048 | 8.82E-01 | 14;657 |
| 0.1 µM EPI | 0.990 | 0.976 | 1.005 | 2.02E-01 | 38;939 | 0.991 | 0.964 | 1.018 | 4.86E-01 | 17;311 | 0.990 | 0.972 | 1.009 | 2.85E-01 | 21;628 |
| 1 µM EPI | 1.004 | 0.995 | 1.014 | 3.47E-01 | 59;1460 | 1.000 | 0.988 | 1.011 | 9.49E-01 | 36;599 | 1.016 | 0.997 | 1.035 | 9.35E-02 | 23;861 |
| COLLAGEN | 0.993 | 0.981 | 1.005 | 2.20E-01 | 71;1814 | 0.995 | 0.981 | 1.009 | 4.73E-01 | 44;744 | 0.988 | 0.968 | 1.009 | 2.65E-01 | 27;1070 |
|  |  |  |  |  |  |  |  |  |  |  |  |  |  |  |  |
| *sample size at different concentrations vary do to concentration titrating scheme originally applied in FHS LTA data acquisition | | | | | | | | | |  |  |  |  |  |  |
| ADP and EPI values are % Maximal Aggregation whereas COLLAGEN is Lag time. | | | | | | |  |  |  |  |  |  |  |  |  |
| Main models according to Metabolic Syndrome (MS) status adjust for age, sex, aspirin usage. Sex stratified models adjusts for age, aspirin usage. | | | | | | | | | | | | |  |  |  |
| HR indicates Hazard ratio; CI95, 95% confidence intervals; P, P value; N, number of events;total at risk. | | | | | | | | |  |  |  |  |  |  |  |

**Supplementary Table 8, Results of Cox Mixed-Effect Hazard Models for Association of Dichotomized Platelet Aggregation Traits with Incident Diabetes and Stratified by Sex**

|  | All | | | | | Male | | | | | Female | | | | |
| --- | --- | --- | --- | --- | --- | --- | --- | --- | --- | --- | --- | --- | --- | --- | --- |
| Model | HR | CI95 low | CI95 high | P | N | HR | CI95 low | CI95 high | P | N | HR | CI95 low | CI95 high | P | N |
| EPI hyper | 0.970 | 0.617 | 1.526 | 8.93E-01 | 153;1473 | 0.957 | 0.461 | 1.987 | 9.04E-01 | 72;561 | 0.983 | 0.554 | 1.747 | 9.54E-01 | 81;912 |
| EPI hypo | 1.018 | 0.524 | 1.978 | 9.56E-01 | 107;817 | 0.753 | 0.314 | 1.805 | 5.16E-01 | 66;476 | 1.632 | 0.562 | 4.741 | 3.58E-01 | 41;341 |
| ADP hyper | 0.828 | 0.519 | 1.320 | 4.18E-01 | 310;2763 | 0.891 | 0.404 | 1.963 | 7.69E-01 | 165;1219 | 0.803 | 0.452 | 1.429 | 4.47E-01 | 145;1544 |
| ADP hypo | 0.963 | 0.464 | 2.001 | 9.18E-01 | 222;1918 | 0.616 | 0.217 | 1.750 | 3.54E-01 | 132;963 | 1.327 | 0.470 | 3.741 | 5.85E-01 | 90;955 |
| Main model adjusts for age, sex, Metabolic Syndrome (MS) status, aspirin usage. Sex stratified models adjusts for age, MS status, aspirin usage. | | | | | | | | | | | | | |  |  |
| HR indicates Hazard ratio; CI95, 95% confidence intervals; P, P value; N, number of events;total at risk. | | | | | | | | | |  |  |  |  |  |  |

**Supplementary Table 9, Sex Stratified Platelet Cell Count Descriptive Summary Statistics of Self-Reported Diabetes Status and Diabetic Medication Usage in the UK BioBank Cohort**

|  | Male | | | Female | | |  |
| --- | --- | --- | --- | --- | --- | --- | --- |
|  | N | Mean | SD | N | Mean | SD | P value |
| Not Diabetic | 194752 | 238.5 | 53.6 | 246250 | 265.7 | 58.5 | 0 |
| All Diabetics | 13661 | 234.9 | 57.5 | 9040 | 271.9 | 65.1 | 0 |
| Metformin | 7772 | 238.8 | 58.2 | 4703 | 280.9 | 65.8 | 1.40E-252 |
| Insulin | 2580 | 239.9 | 60.6 | 1756 | 273.6 | 67.6 | 2.39E-57 |
| Sulphonylurea | 3192 | 240.7 | 59.7 | 1531 | 282.3 | 68.4 | 6.57E-81 |
|  |  |  |  |  |  |  |  |
| N, sample size; SD, standard deviation; P value, univariate T test P value. Medication usage is based on self-report. | | | | | | | |
| Cell count units are N x 10^9 per liter. | | |  |  |  |  |  |

**Supplementary Table 10, Sex Stratified MPV Descriptive Summary Statistics of Self-Reported Diabetes Status and Diabetic Medication Usage in the UK BioBank Cohort**

|  | Male | | | Female | | |  |
| --- | --- | --- | --- | --- | --- | --- | --- |
|  | N | Mean | SD | N | Mean | SD | P value |
| Not Diabetic | 194752 | 9.2770 | 1.0623 | 246250 | 9.3669 | 1.0851 | 2.04E-160 |
| All Diabetics | 13661 | 9.4641 | 1.1300 | 9040 | 9.4585 | 1.1159 | 0.72 |
| Metformin | 7772 | 9.4495 | 1.1282 | 4703 | 9.4118 | 1.0856 | 0.07 |
| Insulin | 2580 | 9.5499 | 1.1450 | 1756 | 9.5910 | 1.1403 | 0.26 |
| Sulphonylurea | 3192 | 9.4352 | 1.1033 | 1531 | 9.3961 | 1.0846 | 0.26 |
|  |  |  |  |  |  |  |  |
| Medication usage is based on self report. | | |  |  |  |  |  |
| N indicates sample size; SD, standard deviation; P value, univariate T test P value. | | | | | |  |  |
| MPV units in fL (femtoliter) | |  |  |  |  |  |  |
